# Supplementary figures and images for: Validation of HIV-1 MA Shell Structural Arrangements and Env Protein Interactions Predict a Role of the MA Shell in Viral Maturation
Source: Viruses. 2023 Mar 30;15(4):893. doi: 10.3390/v15040893 (PMC10144363; doi:10.3390/v15040893)

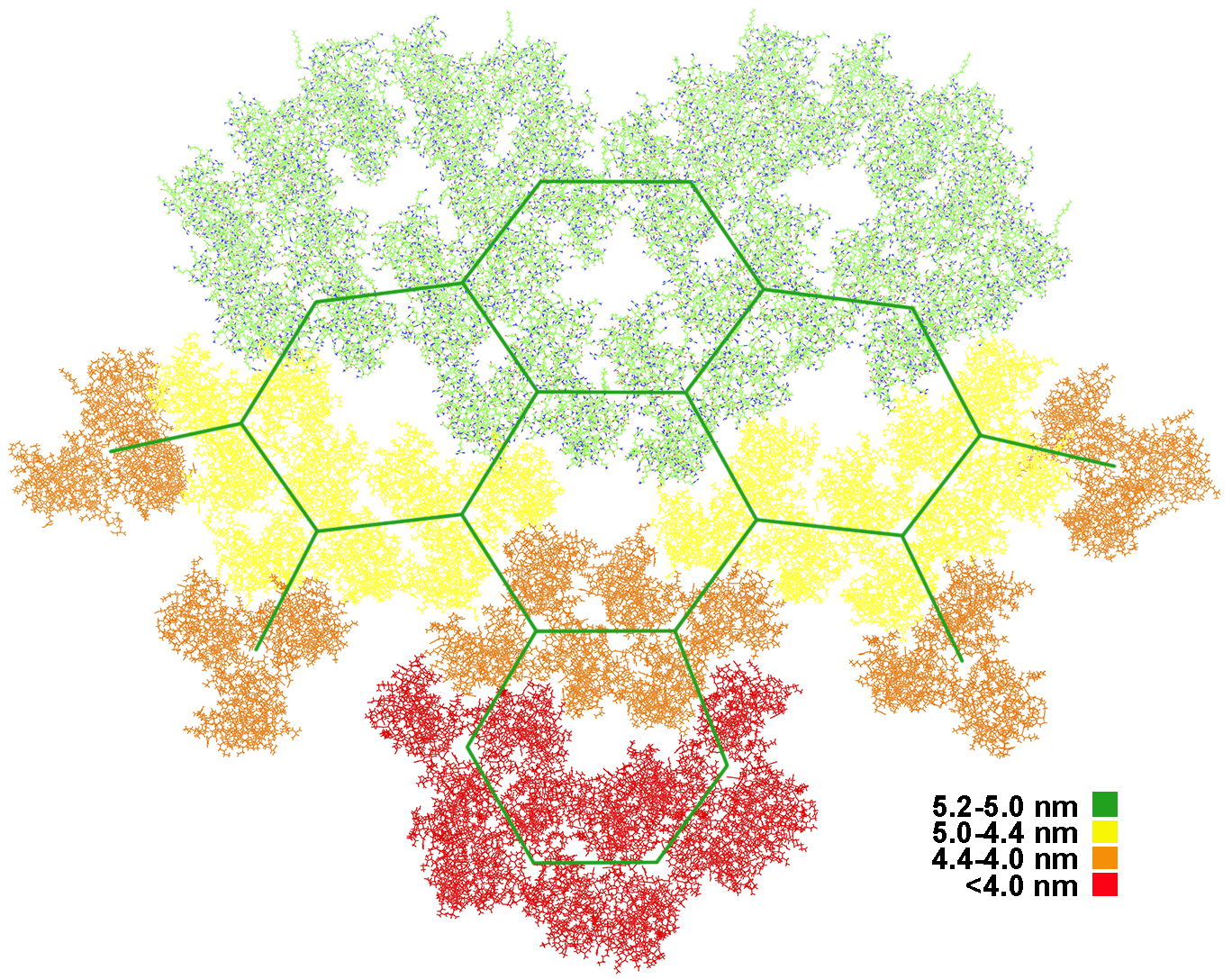

Supplement: Supplementary file 1 [file viruses-15-00893-s001.zip › Supfig1.tif]

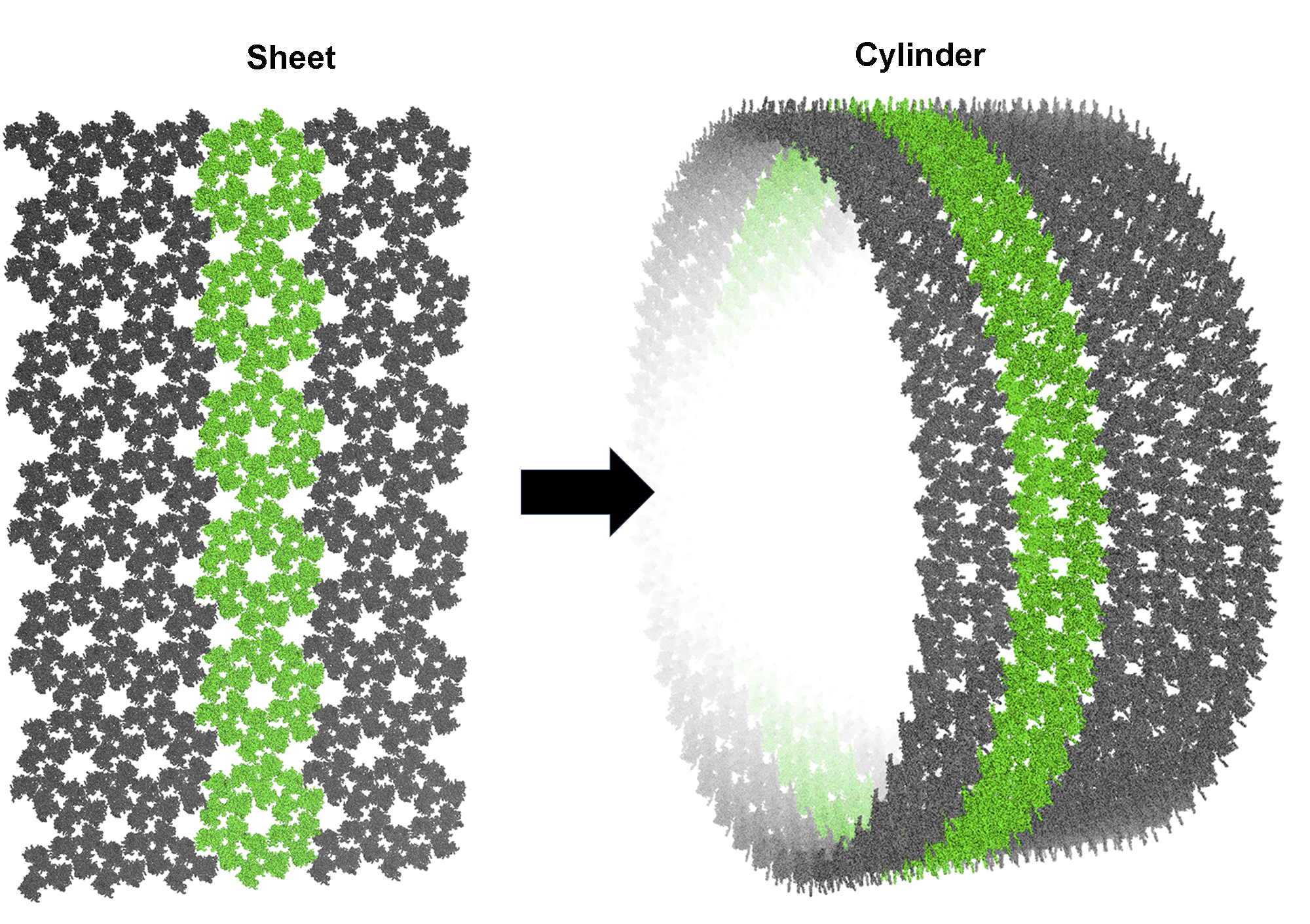

Supplement: Supplementary file 1 [file viruses-15-00893-s001.zip › SupFig2.tif]
